# Transcriptome Analysis Revealed Potential Neuro-Immune Interaction in Papillary Thyroid Carcinoma Tissues

**DOI:** 10.3390/diseases11010009

**Published:** 2023-01-04

**Authors:** Haibei Hu, Qiang Chen, Siqi Zheng, Shan Du, Siqin Ding, Yongzhi Lun

**Affiliations:** 1 Department of Thyroid and Breast Surgery, Shenzhen Hospital (Guangming), University of Chinese Academy of Sciences, Shenzhen 518107, China; 2 Key Laboratory of Medical Microecology, School of Pharmacy and Medical Technology, Putian University, Putian 351100, China; 3 Department of Child Healthcare, Shenzhen Guangming Maternity & Child Healthcare Hospital, Shenzhen 518107, China; 4 Department of Pathology, Shenzhen Hospital (Guangming), University of Chinese Academy of Sciences, Shenzhen 518107, China

**Keywords:** papillary thyroid carcinoma, GSVA, neurons, tumor microenvironment

## Abstract

Background: A recent study reported that papillary thyroid carcinoma (PTC) was associated with increased adrenergic nerve density. Meanwhile, emerging evidence suggested that tumor-innervating nerves might play a role in shaping the tumor microenvironment. We aimed to explore the potential interaction between neuronal markers and tumor microenvironmental signatures through a transcriptomic approach. Methods: mRNA sequencing was conducted using five pairs of PTC and adjacent normal tissues. The Gene Set Variation Analysis (GSVA) was performed to calculate enrichment scores of gene sets related to tumor-infiltrating immune cells and the tumor microenvironment. The potential interaction was tested using the expression levels of a series of neuronal markers and gene set enrichment scores. Results: PTC tissues were associated with increased enrichment scores of CD8 T cells, cancer-associated fibroblasts, mast cells, and checkpoint molecules. The neuronal marker for cholinergic neurons was positively correlated with CD8 T cell activation, while markers for serotonergic and dopaminergic neurons showed an inverse correlation. Conclusion: Distinct neuronal markers exerted different correlations with tumor microenvironmental signatures. Tumor-innervating nerves might play a role in the formation of the PTC microenvironment.

## 1. Introduction

Thyroid cancer is the most common malignant tumor in the endocrine system. The incidence of thyroid cancer has increased worldwide since 1990. A cancer burden analysis using global cancer registries from 1990 to 2017 estimated that the incidence and mortality of thyroid cancer increased by 169% and 87%, respectively [1]. It was noted that nearly half of thyroid cancer cases (41.73%), thyroid cancer-related deaths (50.92%), and disability-adjusted life years (54.39%) were reported in Southern and Eastern Asia [1]. In China, the age-standardized incidence rate by the world standard population is 10.44/100,000, with a cumulative incidence rate (0–74 years old) of 1.00%. Both incidences were above the world average (6.6/100,000 and 0.68%, respectively. https://gco.iarc.fr/ (accessed on 8 May 2021)). The burden of thyroid cancer is high in China.

Bulk RNA sequencing has been frequently used to identify the differentially expressed genes (DEGs), the gene interaction network, and the signaling pathways/biological processes implicated in cancers. Single-sample gene set enrichment analysis (ssGSEA) or gene set variation analysis (GSVA) uses a gene expression matrix to generate enrichment scores of gene sets (pathways, ontologies, or cell type-specific markers) of interest. Enrichment scores have been normalized across samples and can therefore be used to evaluate the degree of pathway activities or proportions of cell types between samples or groups. Recent studies have successfully employed gene sets of tumor-infiltrating immune cells or tumor microenvironments to identify the subtypes of cancers [2,3,4]. It is also possible to conduct correlation analysis between different gene sets or between genes and gene sets, which allow us to explore the interactions between cell types (cell type-specific gene sets) or the genes responsible for activating cell types. Recently, using immunohistochemistry, it was reported that the tissues of papillary thyroid carcinoma (PTC), the most common type of thyroid cancer, were innervated by increased adrenergic nerve fibers [5]; moreover, the nerve density was associated with malignancy, although the mechanism by which nerve fibers affect PTC pathogenesis was still unclear. Interestingly, emerging evidence suggested that tumor-innervating nerves are not bystanders of tumorigenesis. Instead, they might play roles in the formation of the tumor microenvironment [6,7]. In this study, we aimed to explore the potential effects of neurons on the signatures of the tumor microenvironment by using mRNA profiling of our cohort and the GSVA method.

## 2. Methods and Materials

### 2.1. Patient and Tissue Collection

The study was approved by the clinical research ethics committee at Shenzhen Hospital (Guangming) of the University of Chinese Academy of Sciences, following the Declaration of Helsinki. All participants had signed the informed consent forms. A total of 5 patients diagnosed with PTC were recruited following the inclusion criteria: patients diagnosed with papillary thyroid cancer by fine-needle aspiration, and no radiotherapy or chemotherapy before surgery. The exclusion criteria are patients diagnosed with papillary thyroid carcinoma combined with other tumors, and/or with a history of radiotherapy, chemotherapy, or radioactive exposure. Tissues were collected during surgery. The tumor tissues and the paired normal tissues adjacent to the tumors were then dissected by a pathologist for RNA sequencing. Demographics and TNM staging were demonstrated in Table 1.

### 2.2. RNA Sequencing Analysis

Total RNA was extracted using RNeasy Midi kits (QIAGEN, Shenzhen, China), followed by library preparation with Illumina Stranded mRNA prep kits (Illumina, San Diego, CA, USA) and mRNA sequencing on the NextSeq 2000 system (Illumina). Briefly, after treatment with DNase I, 1 µg of total RNAs were fragmented and converted into cDNAs, which were then used for adapter tagging, size selection, and sequencing to generate paired-end reads. For RNA-seq data preprocessing, Trimmomatic was used to trim adaptor sequences, remove low-quality reads, and eliminate low-quality bases [8]. The clean reads were then aligned with the Homo sapiens GRCh38 reference genome using STAR [9]. The raw counts of genes were generated using featureCounts [10]. The genes that have an average raw count greater than 10 across all samples were included for the identification of differentially expressed genes (DEGs) using DESeq2 [11]. The top 200 upregulated or top 200 downregulated DEGs were then analyzed for gene ontologies (GO Biological process 2021) and cell type mapping (Descartes cell types and tissues 2021) with Enrichr [12]. The GO terms were clustered using the Leiden algorithm [13] and plotted on the first two UMAP dimensions (https://doi.org/10.48550/arXiv.1802.03426, accessed on 8 May 2021). An online tool for plotting is available on Appyters (https://appyters.maayanlab.cloud/#/Enrichment_Analysis_Visualizer, accessed on 8 May 2021). The script for this online tool can be found on github (https://github.com/MaayanLab/Enrichr-Viz-Appyter/blob/master/Enrichr-Processed-Library-Storage/Scatterplot/scatter_libs.py, accessed on 8 May 2021) The 30 most dysregulated DEGs with an adjusted *p* value less than 0.05 were plotted using ggplot2. All the software and packages used in this study were summarized in Supplementary Material S1. The RNAseq data that support the findings of this study are openly available in the Gene Expression Omnibus (GEO) database (accession number: GSE201365).

### 2.3. Gene Set Enrichment Analysis (GSEA)

Normalized counts generated by DESeq2 were used for GSEA [14]. The gene set permutation method was adopted and the number of permutations was set as 1000. The GO terms with false discovery rate (FDR) adjusted *p*-values less than 0.05 were plotted using ggplot2.

### 2.4. Gene Set Variation Analysis (GSVA)

The GSVA method was used to calculate the enrichment scores of 28 tumor-infiltrating immune cell signatures [15] and 28 tumor microenvironment-related gene signatures [4]. The enrichment scores of all samples were plotted using the ggcorrplot R package. The difference between the groups (adjacent normal and PTC tissues) was compared using a paired *t*-test. The correlation between the enrichment score of specific gene set and the expression level of a specific gene (in TPM, transcripts per million) was analyzed using Spearman’s rank correlation method.

## 3. Results

### 3.1. DEGs Identification and Gene Ontology Analysis

**Figure 1:**
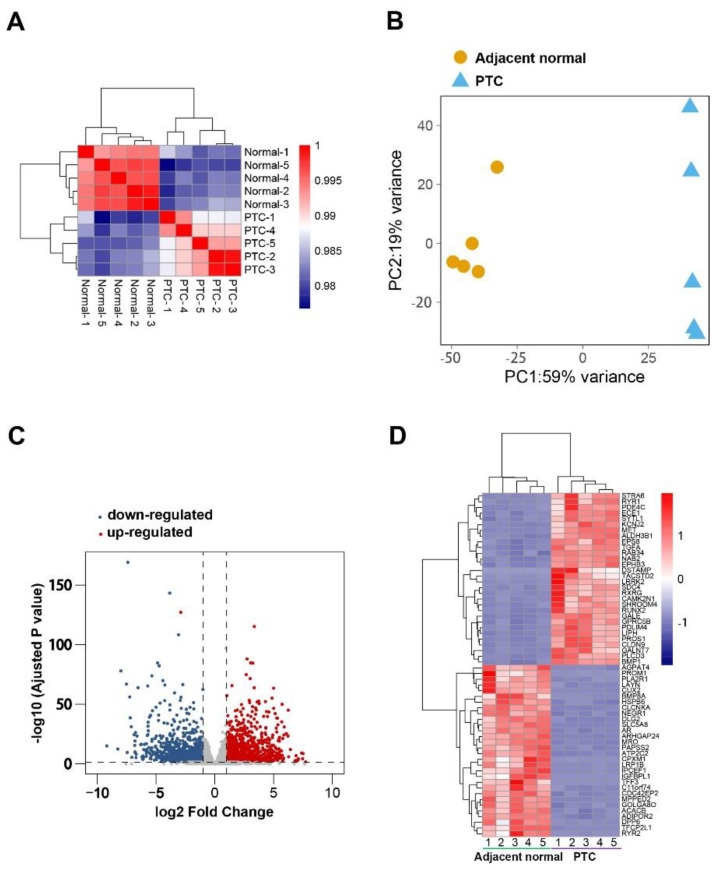
DEGs identification. (**A**) Pearson’s correlation of samples. (**B**) PCA of samples. (**C**) Volcano plot of gene expression. (**D**) Heatmap of top 30 upregulated and top 30 downregulated DEGs.

**Figure 2:**
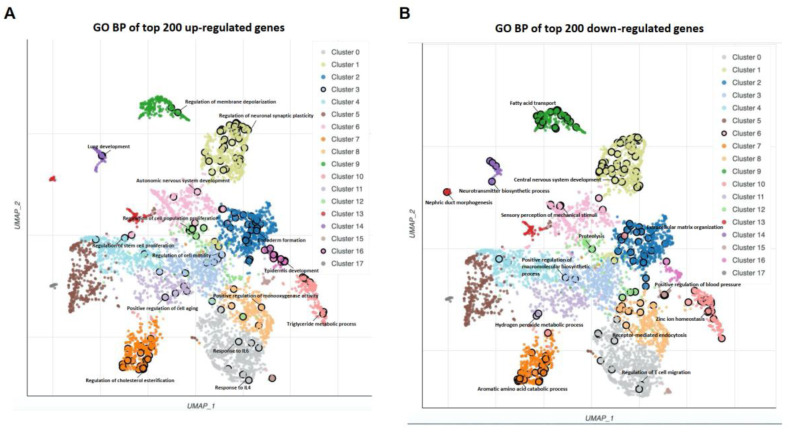
Gene ontology analysis. (**A**) GO biological processes of the top 200 upregulated DEGs. (**B**) GO biological processes of the top 200 downregulated DEGs.

We used matrices of logarithmically transformed normalized counts to perform a correlation analysis between samples. Unsupervised clustering demonstrated that normal samples (adjacent normal tissues) and tumor samples (PTC tissues) were clearly separated (Figure 1A). This was consistent with principal components analysis (Figure 1B). DESeq2 identified 2673 DEGs with absolute fold changes equal to or greater than 2 (adjusted *p* value < 0.05), of which 1405 DEGs were upregulated and 1268 were downregulated in PTC tissues compared to normal tissues (Figure 1C). The top 30 upregulated and top 30 downregulated genes were plotted (Figure 1D). Since the dysregulated genes with higher fold changes may play a more significant role in pathogenesis, we performed a biological process (BP) analysis of gene ontology (GO) using the top 200 gene lists. This generated hundreds of GO terms (Supplementary Material S2), which were then clustered according to their similarity and plotted (Figure 2A,B). The representative biological processes have been marked in Figures. For the top 200 upregulated genes, the cancer-related terms such as “regulation of cell population proliferation”, “positive regulation of cell aging”, and “regulation of cell motility” were highlighted. In addition, biological processes associated with the immune response, including “response to IL4”, “response to IL6”, and “positive regulation of monooxygenase activity” have been identified. Interestingly, several terms related to nervous system, including “autonomic nervous system development”, “regulation of neuronal synaptic plasticity”, and “regulation of membrane depolarization” have also been enriched. Similarly, the top 200 downregulated genes were enriched for several nervous-system-related biological processes, such as “central nervous system development”, “neurotransmitter biosynthesis process”, and “sensory perception of mechanical stimuli”. These results implied that abnormal neuronal events may play a role in PTC pathogenesis.

### 3.2. GSEA

**Figure 3:**
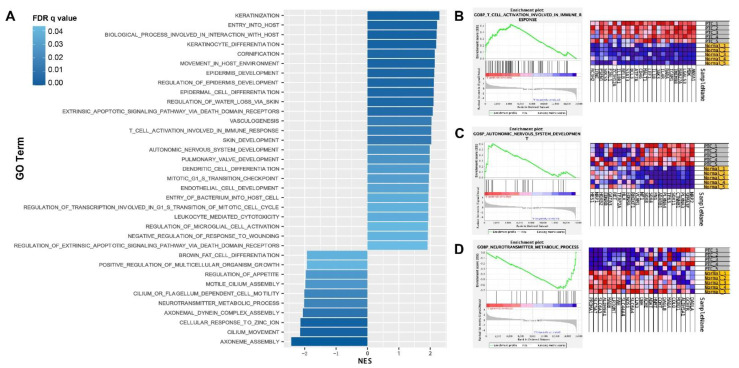
GSEA. (**A**) GO terms enriched by PTC tissues (NES > 0) or adjacent normal tissues (NES < 0). (**B**–**D**) Representative enrichment plots and heatmap of genes involved in selected GO terms.

Enrichment analysis using the DEGs list neglects the fact that gene alterations of different degrees can have different weights on the modulation of biological processes or signaling pathways. To this end, we performed GSEA, which employed a ranked gene list for ontologies annotation. The gene sets of the GO biological processes (version 7.4) were used. As demonstrated in Figure 3A, 25 biological processes were enriched in PTC tissues (normalized enrichment score, NES > 0, FDR adjusted *p* < 0.05), while 10 occurred in normal tissues (NES < 0, FDR adjusted *p* < 0.05). As expected, several ontologies involved in “hallmarks” of cancers were highlighted, including “vasculogenesis”, “mitotic G1 S transition checkpoint”, and “intrinsic apoptotic signaling pathway via death domain receptors”. In addition, the increased NES of “dendritic cell differentiation”, “T cell activation involved in immune response”, and “leukocyte mediated cytotoxicity” indicated that altered adaptive immune responses might play a role in PTC development (Figure 3A). The enrichment plot of T cell activation and a part of genes with high variance between groups were demonstrated (Figure 3B). Finally, in accordance with the top 200 GO analysis, nerve-related terms were also identified, including “autonomic nervous system development”, “axoneme assembly”, “axonemal dynein complex assembly”, and “neurotransmitter metabolic process” (Figure 3A).

### 3.3. GSVA of the Signatures of Tumor-Infiltrating Immune Cells and Tumor Microenvironment

**Figure 4:**
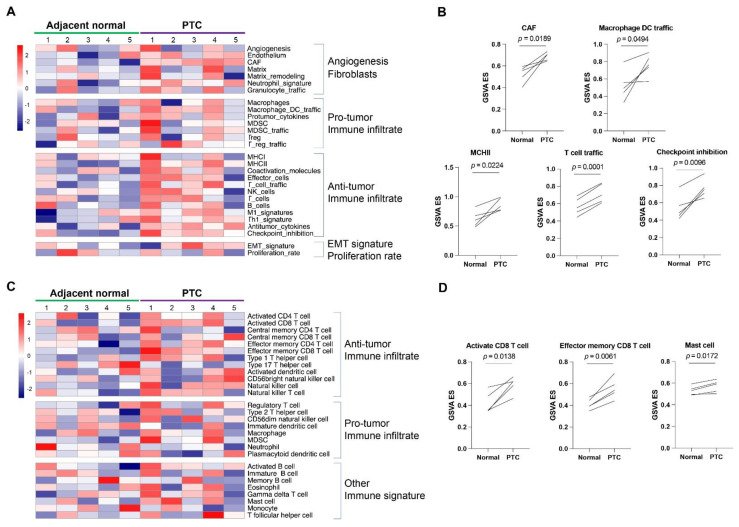
GSVA of the signatures of tumor infiltrating immune cells and tumor microenvironment. (**A**) Heatmap of enrichment scores of 28 tumor microenvironment-related gene sets. (**B**) The gene sets with significant difference between the groups. (**C**) Heatmap of enrichment scores of gene sets of 28 tumor-infiltrating immune cells. (**D**) The gene sets with significant difference between groups. Paired *t*-tests were conducted.

Enrichr and GSEA implicated dysregulated immune responses in PTC tissues. To further delineate the altered microenvironment of PTC tissues and the profiling of tumor infiltrating immune cells, a GSVA was performed. An analysis of 28 tumor microenvironment-related gene sets suggested that the PTC tissues were enriched with the signatures of “CAF (Cancer associated fibroblasts)”, “Macrophage DC traffic”, “MHCII”, and “T cell traffic” compared to adjacent normal tissues (Figure 4A,B, paired *t*-test). This result was consistent with previous GSEA results in Figure 3 and highlighted the involvement of adaptive immune responses. It was noted that “checkpoint inhibition” was also enriched in PTC tissues, indicating immune escape (Figure 4B, paired *t*-test). Although there was no significant difference in PDCD1 and PD-L1 expression levels between PTC and adjacent normal tissues, PDCD1 was decreased in 3 of 5 PTC tissues (Supplementary Material S3). On the other hand, PD-L1 was increased in 3 of 5 PTC tissues (Supplementary Material S3). An analysis of 29 tumor-infiltrating cell gene sets identified that two gene sets associated with CD8 T cells and one gene set with mast cell signatures were significantly enriched in PTC tissues compared to adjacent normal tissues (Figure 4C,D, paired *t*-test).

### 3.4. Correlation of Neuronal Markers and Immune Signatures

**Figure 5:**
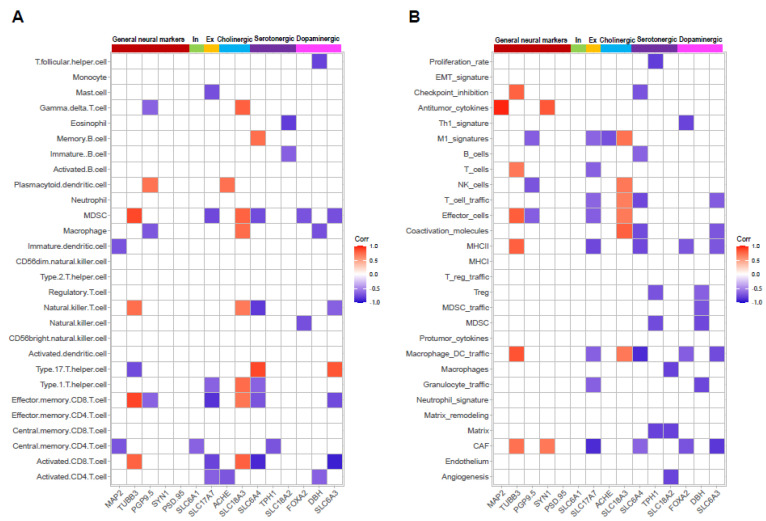
Correlation of neuronal markers and immune signatures. (**A**,**B**) The correlation between enrichment scores of 28 tumor-infiltrating immune cells (**A**), 28 tumor microenvironment-related gene sets (**B**), and TPM values of neuronal markers. The significant correlations (*p* < 0.05, Pearson’s correlation) were demonstrated as a gradient of red (positive) or blue (negative) according to the coefficients.

Recent studies suggested that tumor-innervating neurons are not bystanders of tumor development [16,17]. Instead, these neurons may play a role in the microenvironment of tumor tissues [7]. We asked whether neuronal marker gene expression levels correlate with tumor microenvironment signatures. The TPM values of marker genes, including general neuronal markers (MAP2, TUBB3, PGP9.5, SYN1, and PSD.95), and the markers for inhibitory neurons (SLC6A1), excitatory neurons (SLC17A7), cholinergic neurons (ACHE, SLC18A3), Serotonergic neurons (SLC6A4, TPH1, SLC18A2), as well as dopaminergic neurons (FOXA2, DBH, SLC6A3), were plotted to the enrichment scores (ES) of gene sets. Interestingly, the common axonal marker TUBB3 was positively correlated with the ES of activated CD8 T cells, effector memory CD8 T cells, and natural killer T cells (Figure 5A). A similar association was also identified between the cholinergic neuronal marker SLC18A3 (Figure 5A). However, negative correlations were found between these three gene sets and markers for serotonergic or dopaminergic neurons (Figure 5A), implying that different types of neurons might be associated with differential immune responses.

In terms of tumor microenvironment signatures, the general marker TUBB3 was positively correlated with gene sets involved in both pro-tumor (e.g., CAF and Macrophage DC traffic,) and anti-tumor (e.g., MCHII, Effector cells, T cells, and checkpoint inhibition) actions (Figure 5B). The markers for neuronal subtypes, on the other hand, exerted variant associations with these gene sets. More specifically, cholinergic neuronal marker SLC18A was positively correlated with multiple gene sets responsible for anti-tumor effects, while serotonergic and dopaminergic neuronal markers showed inverse correlations (Figure 5B).

## 4. Discussion

The present study compared the mRNA profiles of PTC tissues and the paired adjacent normal tissues. GO analysis using a chi-square test or GSEA methods suggested that PTC tissues were characterized with abnormal neuron-related biological processes and cytotoxic T-cell-related immune responses. We further performed GSVA to explore the potential alterations in the tumor microenvironment by mapping the expression profile to gene sets regarding tumor-infiltrating immune cells and signatures of the microenvironment. We found that PTC tissues were associated with higher enrichment scores of T-cell-related signatures and several gene sets with pro-tumor features (such as CAF, macrophage DC traffic and checkpoint inhibition); moreover, expression levels of markers for cholinergic neurons were positively correlated with the enrichment scores of T-cell-related gene sets, while markers for serotonergic and dopaminergic neurons showed inverse correlations. We concluded that PTC pathologies were characterized by potential alterations in neural biological processes that might play a role in the formation of the PTC microenvironment.

Previous transcriptome studies had revealed significantly increased overall immune levels in PTC tissues compared to normal tissues [18,19,20]. The signatures of both anti-tumor and tumor-promoting immune cells were evident in PTC tissues. CD8 T cells are considered anti-tumor infiltrating cells leading to inhibition of cancer cell growth and apoptosis. A higher degree of CD8 T cell infiltration predicted better outcomes in patients with PTC [21,22]. CD8 T cells were overall found to be decreased in PTC tissues from TCGA and several GEO datasets compared to normal tissues. However, our studies demonstrated the enrichment scores of two CD8 T-cells-related gene sets, namely activated CD8 T cells and effector memory CD8 T cells, which were higher in PTC tissues than in paired adjacent normal tissues. The different clinical staging could be attributed to the inconsistent findings. The TCGA and other GEO datasets recruited patients diagnosed with PTC at various TNM stages, whereas all patients in the current study were classified as T1 stage. It has been reported that higher levels of CD8 T cells were observed in PTC tissues from patients at stage I and II than in other stages [18]. It is likely that the number of CD8 T cells gradually decreases during PTC progression. Mast cells are resident immune cells in vascularized tissues. Activated mast cells are important sources of proinflammatory mediators, including lipid mediators, cytokines, chemokines, and angiogenic factors [23], thereby implying that mast cells could be active players in various diseases. In fact, the tumor-promoting roles of mast cells (tumor-associated mast cells) has been repeatedly reported [24,25]. For example, in PTC xenografts models, co-injection with mast cells significantly facilitated the growth of thyroid cancer cells in vivo [26]. Besides mast cells, CAF is another type of tissue resident cell (stromal cell) involved in tumorigenesis and cancer progression. An analysis of TCGA demonstrated that PTC tissues had significantly higher CAF-associated signatures than normal tissues, which also correlated positively with tumor-promoting immune cell infiltration [27]. Histological studies also showed that CAF appeared in the invasive front of cancer tissues, supporting its role in promoting PTC [28]. In accordance with previous studies, our study suggested that PTC tissues were associated with higher enrichment scores of mast cells and CAF than paired normal tissues. These results suggested that mRNA profiling is a sensitive approach for exploring the cellular network in the PTC microenvironment. A correlation analysis could be applied to discover the potential interaction between cell types and shed light on a novel strategy to control tumor-promoting cells in microenvironment.

The identification of neural signatures was another interesting finding of the current study. GO analysis using the top 200 (upregulated or downregulated) DEGs or entire expression matrix revealed potential alterations in nervous-system-related biological processes in PTC tissues. In particular, “autonomic nervous system development” was highlighted in PTC when using the GSEA method. The autonomic system consists of the sympathetic nervous system and parasympathetic nervous system, in which adrenergic (dopaminergic) and cholinergic neurons are typical neuronal types, respectively. Interestingly, a recent study reported that nerve density was significantly increased in PTC tissues compared to benign thyroid [5]. Meanwhile, markers for different types of neurons, namely adrenergic, cholinergic, and peptidergic neurons, could be detected, thereby indicating that different types of neurons are present in the PTC tissues. Our mRNA profiling study also confirmed the expression of several general neuronal markers (TUBB3) and cell type specific markers (cholinergic, dopaminergic, and serotonergic neurons); moreover, we found that different neuronal markers had opposite correlations with the enrichment scores of tumor microenvironment-related gene sets, implying that distinct neuronal types might play different modulating roles in microenvironment formation. For example, the cholinergic marker SLC18A3 was positively correlated with the enrichment scores of “activated CD8 T cells”, “effector memory CD8 T cells”, and “T cell traffic”, while dopaminergic marker SLC6A3 showed inverse correlations. Previous functional studies provided evidence that these correlations could be cause-and-effect relationships. The neurotransmitter of cholinergic neurons, i.e., acetylcholine, appears to be critical for the differentiation of CD8 T cells into cytolytic T lymphocytes, while knockout of acetylcholine receptors (muscarinic receptors) leads to a defect in CD8 T cell differentiation [29,30]. On the other hand, the neurotransmitter of adrenergic neurons, norepinephrine, could inhibit the effector function of CD8 T cells by binding to β2-adrenergic receptor (mainly expressed in the activated and memory CD8 T cells), thus blocking the metabolic reprogramming of these cells [31]. Similar findings have been reported elsewhere [32]. Therefore, our study indicated that tumor resident neuronal cells might differentially regulate the tumor microenvironment. Neural modulation, such as vagus nerve stimulation, could be adopted as an adjuvant approach to promote immune therapy of cancer. Nonetheless, the interesting findings of the current study were obtained by in silico analysis on a small sample size. Further histological and immunophenotyping studies need to be performed to consolidate the relationship between nerve fibers and the PTC immune microenvironment in the future. Animal studies are also favorable to prove causality.

## 5. Conclusions

mRNA profiling is useful to reveal the cellular network in PTC tissues. The nervous system might play a role in the formation of the PTC immune microenvironment.

## Figures and Tables

**Table 1:** Patients’ characters.

Sample ID	Gender	Age	TNM Staging	Clinical Stage
1	Female	39	T1bN0M0	I
2	Female	22	T2N1aM0	I
3	Male	55	T1aN1aM0	III
4	Female	24	T1bN1aM0	I
5	Male	43	T1bN1aM0	I

## Data Availability

The RNAseq data that support the findings of this study are openly available in the GEO database (accession number: GSE201365).

## Supplementary Materials

The following supporting information can be downloaded at: https://www.mdpi.com/article/10.3390/diseases11010009/s1, Supplementary Matrial S1: Software and packages used in this study. Supplementary Material S2: GO terms; Supplementary Material S3: PDCD1 and PD-L1 expression levels.
