# Supplementary material for: Transcriptome Analysis Revealed Potential Neuro-Immune Interaction in Papillary Thyroid Carcinoma Tissues
Source: Diseases. 2023 Jan 4;11(1):9. doi: 10.3390/diseases11010009 (PMC9844349; doi:10.3390/diseases11010009)
Supplement: Supplementary file 1 [file diseases-11-00009-s001.zip › Supplementary Material S1.pdf]

Table 2. Software and packages used in this study.

Methods	References	Link
**Trimmomatic**	Bolger, Lohse et al. 2014	
**STAR**	Dobin, Davis et al. 2013	
**FeatureCounts**	Liao, Smyth et al. 2014	
**DESeq2**	Love, Huber et al. 2014	
**Enrichr**	Xie, Bailey et al. 2021	
**Leiden algorithm**	Traag, Waltman et al. 2019	https://doi.org/10.48550/arXiv.1802.03426
**Appyters**		https://appyters.maayanlab.cloud/#/Enrichment_Analysis_Visualizer
**Github**		https://github.com/MaayanLab/Enrichr-Viz-Appyter/blob/master/Enrichr-Processed-Library-Storage/Scatterplot/scatter_libs.py
**Gene Set Enrichment Analysis (GSEA)**	Subramanian, Tamayo et al. 2005	
**Gene Set Variation Analysis (GSVA)**	Charoentong, Finotello et al. 2017<br>Bagaev, Kotlov et al. 2021	
