# Supplementary figures and images for: Transcriptome Analysis Revealed Potential Neuro-Immune Interaction in Papillary Thyroid Carcinoma Tissues
Source: Diseases. 2023 Jan 4;11(1):9. doi: 10.3390/diseases11010009 (PMC9844349; doi:10.3390/diseases11010009)

A

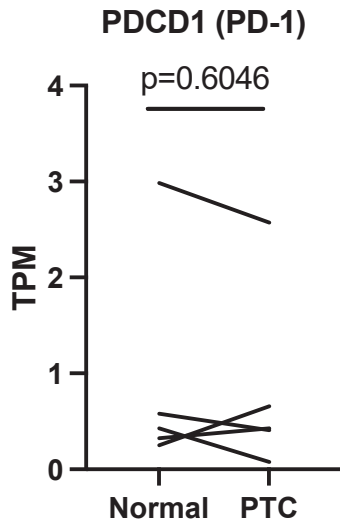

B

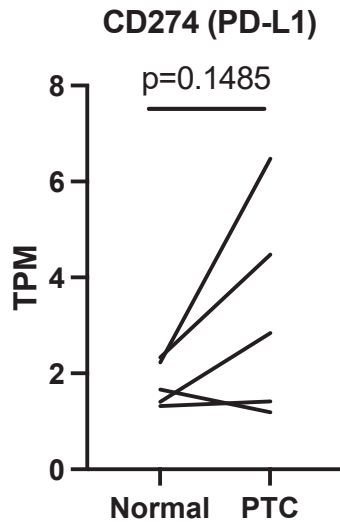

Supplement: Supplementary file 1 [file diseases-11-00009-s001.zip › Supplementary Material S3.pdf]
